# Automated alignment-based curation of gene models in filamentous fungi

**DOI:** 10.1186/1471-2105-15-19

**Published:** 2014-01-16

**Authors:** Ate van der Burgt, Edouard Severing, Jérôme Collemare, Pierre JGM de Wit

**Affiliations:** 1 Laboratory of Phytopathology, Wageningen University & Research Centre, P.O. Box 16, 6700 AA Wageningen, The Netherlands; 2 Applied Bioinformatics, Plant Research International, Wageningen University & Research Centre, P.O. Box 16, 6700 AA Wageningen, The Netherlands; 3 Laboratory of Genetics, Wageningen University & Research Centre, P.O. Box 16, 6700 AA Wageningen, The Netherlands

**Keywords:** Gene model, Automated gene model curation, Sequence error, Truncated gene model, Pseudogene, Fungal genome, *Cladosporium fulvum*

## Abstract

**Background:**

Automated gene-calling is still an error-prone process, particularly for the highly plastic genomes of fungal species. Improvement through quality control and manual curation of gene models is a time-consuming process that requires skilled biologists and is only marginally performed. The wealth of available fungal genomes has not yet been exploited by an automated method that applies quality control of gene models in order to obtain more accurate genome annotations.

**Results:**

We provide a novel method named alignment-based fungal gene prediction (ABFGP) that is particularly suitable for plastic genomes like those of fungi. It can assess gene models on a gene-by-gene basis making use of informant gene loci. Its performance was benchmarked on 6,965 gene models confirmed by full-length unigenes from ten different fungi. 79.4% of all gene models were correctly predicted by ABFGP. It improves the output of *ab initio* gene prediction software due to a higher sensitivity and precision for all gene model components. Applicability of the method was shown by revisiting the annotations of six different fungi, using gene loci from up to 29 fungal genomes as informants. Between 7,231 and 8,337 genes were assessed by ABFGP and for each genome between 1,724 and 3,505 gene model revisions were proposed. The reliability of the proposed gene models is assessed by an *a posteriori* introspection procedure of each intron and exon in the multiple gene model alignment. The total number and type of proposed gene model revisions in the six fungal genomes is correlated to the quality of the genome assembly, and to sequencing strategies used in the sequencing centre, highlighting different types of errors in different annotation pipelines. The ABFGP method is particularly successful in discovering sequence errors and/or disruptive mutations causing truncated and erroneous gene models.

**Conclusions:**

The ABFGP method is an accurate and fully automated quality control method for fungal gene catalogues that can be easily implemented into existing annotation pipelines. With the exponential release of new genomes, the ABFGP method will help decreasing the number of gene models that require additional manual curation.

## Background

In the past decade, numerous fungal genomes of importance to medicine, agriculture and industry have been sequenced [1,2] and continuous innovations in next generation sequencing technology will spur this number to rapidly increase further. Once sequenced and assembled, genomes are annotated through an automated gene-calling pipeline, which is still an error-prone process, particularly for the highly plastic and diverse genomes of fungal species.

Most gene annotation pipelines integrate different gene prediction algorithms to increase the accuracy of the annotation [3]. These algorithms include *ab initio* supervised, *ab initio* unsupervised and (supervised) alignment-based gene predictors, which are implemented in tools such as Augustus [4], GeneMark-ES [5] and TWINSCAN 2.0α [6], respectively. Augustus is one of the most frequently employed and best performing *ab initio* supervised gene prediction tools that offers parameterizations for several dozens of fungi [4]. For species lacking a provided parameterization, a considerable manual input is required to obtain such species-specific parameterization by training the algorithm with a large sample (~1000) of correct gene models [5]. Thus, its applicability is limited to only those species for which parameterization is available [5,6]. GeneMark-ES-2 is an *ab initio* unsupervised gene predictor iteratively training itself on the input genome sequence alone that outperformed Augustus [5], but is reported to be relatively inaccurate in predicting single exon genes [5]. A hybrid strategy between *ab initio* and alignment- (or evidence) based gene prediction is currently implemented in several tools. Updated versions of Augustus integrate evidence obtained from unigene alignments [4], protein multiple sequence alignments [7] and intron- and exon-hints acquired from RNA-Seq data, which greatly improved their prediction accuracy. To our knowledge, alignment-based gene prediction in fungi using genomic data alone has only been successfully applied using TWINSCAN 2.0 α, which was specifically adapted and trained to *Cryptococcus neoformans*[6]. In that case, the whole-genome DNA alignment of two strains of this fungus, whose genomes are largely syntenic and exhibit around 95% nucleotide identity in coding regions, served as input. The reported ~60% gene accuracy clearly outperformed non-alignment-based *ab initio* gene prediction software [6]. TWINSCAN 2.0 α requires extensive species-specific training and parameterization, offering a tailor-made solution for a defined pair of related species only. Most importantly, the approach taken in TWINSCAN 2.0 α is difficult to apply to fungal genomes because of their high plasticity [8-10]. The absence of conserved regions exhibiting macro- or even meso-synteny between related fungal genomes [8] severely hampers the construction of whole-genome DNA alignments. Besides reshuffled gene orders, a highly variable gene content is also observed among fungi with a large number of genes showing a discontinuous distribution in the fungal tree of life. This is caused by frequent gene, gene-cluster, segmental and whole chromosome duplications, losses or horizontal transfers, which have created complex variation in both gene family expansion and reduction [8,10]. Although homologous gene loci can often be inferred easily between distantly related fungi, annotation of fungal genomes by classical alignment-based gene prediction tools is problematic. In recent years, *ensemble* predictors have been developed to weigh and combine similarity evidence and the predictions made by various other tools into a single, more accurate gene model [11,12]. However, it “often requires significant effort in implementation to cast comparative information into a form compatible with the existing gene models” [13].

Because none of the available gene prediction tools were specifically developed for fungal genomes, automatic gene annotation of fungi often yields a relatively high fraction of incorrect gene models. These can only be revised through a time-consuming process of quality control and manual curation by skilled biologists or bioinformaticians, but this is often only marginally performed. Manual curation usually involves comparative analyses with tools that can accurately identify a spliced gene structure in a target DNA sequence using a homologous protein sequence as a so-called “informant” sequence (e.g. GeneWise [14], Scipio [15], etc.). However, a large proportion of gene models and derived protein sequences in current fungal sequence releases contain errors, and a manual curator can easily propagate existing errors when using incorrectly predicted informant protein(s). A typical example of the marginal quality of fungal gene catalogues is exemplified by the re-annotation of the *Fusarium graminearum* genome [16]. In the new version, 1,770 gene models were revised by using various new gene predictors, exploiting expression data, performing extensive manual curation and evidence-based selection of the best gene model from alternative predictions [16]. Despite this effort, recent RNA-Seq data provided experimental proof for at least another 655 incorrectly predicted gene models in the latest version of the *F. graminearum* annotation [17].

We have now entered an era in which genome sequencing of clusters of related fungi will be performed on a massive scale. Subsequent gene prediction on these genomes will require automation with very little manual inspection [6]. Although gene prediction software suitable for fungal genomes has become more accurate over the last decade, they are still error-prone. A method that facilitates or automates the process of curating gene models is therefore needed to increase the accuracy of the catalogues of predicted genes in sequenced fungal genomes. Here, we present a novel gene-by-gene method for alignment-based gene prediction that is particularly suitable for the plastic genomes of fungi. Our method, called alignment-based fungal gene prediction (ABFGP), (i) provides improved accuracy of predicted gene models, (ii) is species-independent, (iii) does not require partial or whole-genome DNA alignments, (iv) does not require supervision and (v) can use a variable number of informant genes. We demonstrate the accuracy and versatility of the ABFGP method by re-annotating the genomes of a selection of six sequenced Ascomycete fungi.

## Results

### The alignment-based fungal gene prediction (ABFGP) method

**Figure 1:**
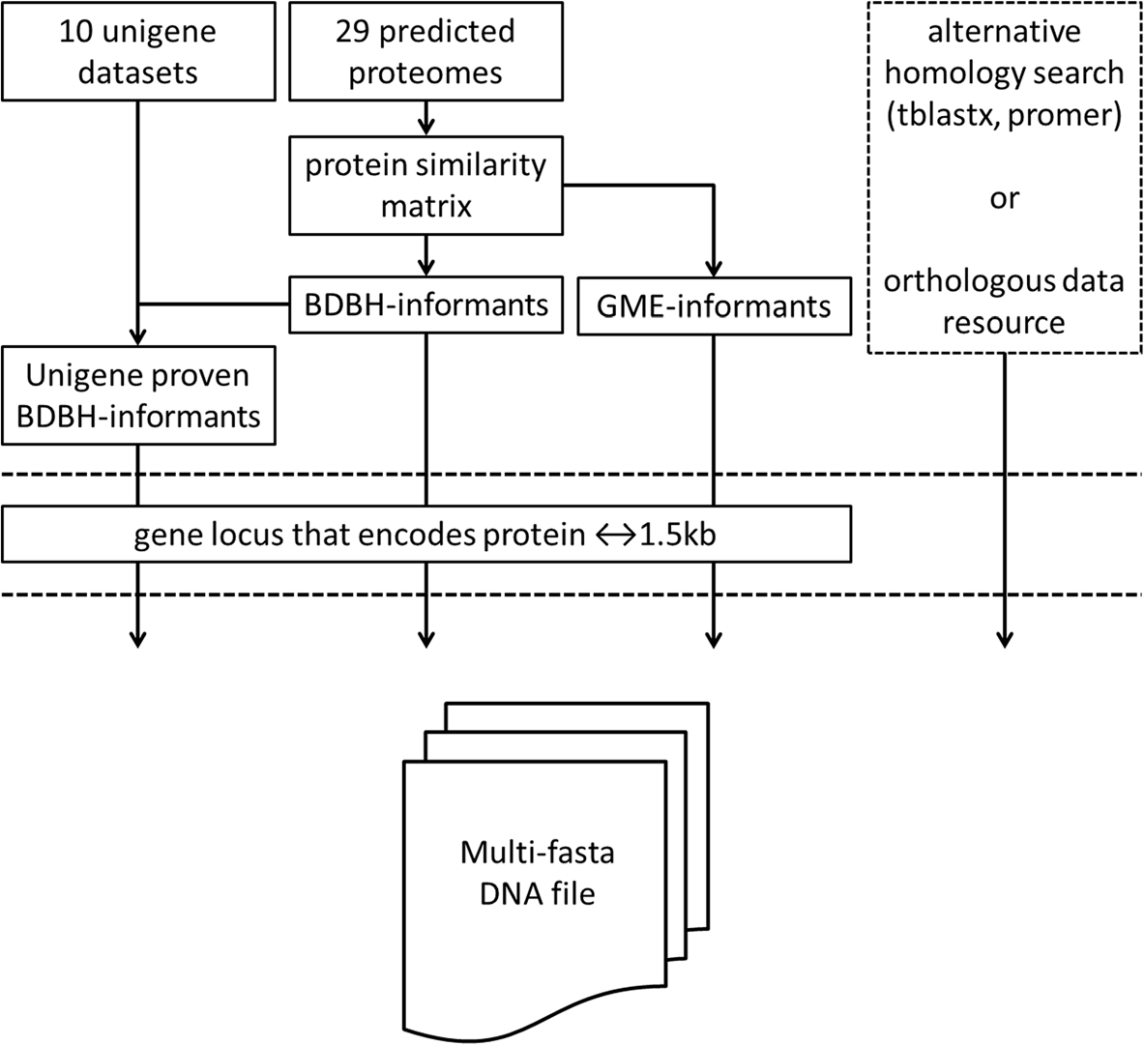
Flow diagram of informant gene selection for the alignment-based fungal gene prediction (ABFGP) method.

The ABFGP method re-annotates gene models on a gene-by-gene basis by using informants, which differ from regular alignment-based approaches that require a whole-genome DNA alignment. An ABFGP informant refers to the genomic locus at which an homologous gene is encoded that may support revision of the target gene locus. First, a similarity matrix of predicted protein sequences from several fungal species is obtained (Figure 1; Additional file 1). From this matrix, bi-directional best hits (BDBH) with sufficient overlap between both annotated proteins are selected, representing most likely orthologous informant gene loci. Subsequently, the genomic loci that encodes these proteins - not the predicted proteins themselves - are used as informants to avoid propagation of errors in the gene structures. Other resources can be used to find informant gene loci such as unigene datasets or any alternative homology search (Figure 1).

**Figure 2:**
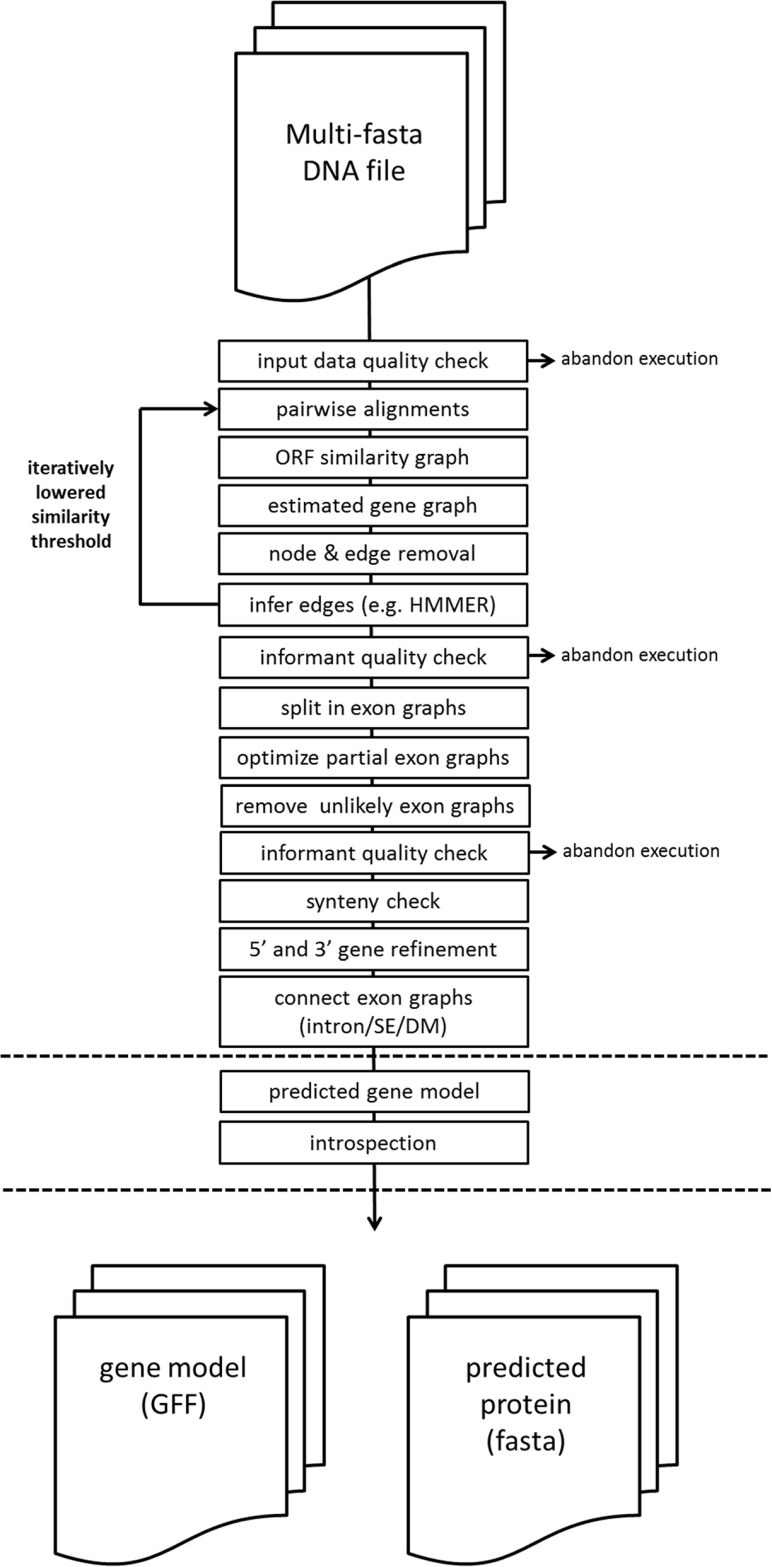
Flow diagram of the ABFGP method.

The ABFGP method is an automated workflow that includes all steps typically undertaken when performing manual annotation of a predicted gene model. It comprises nucleotide and protein similarity searches (BLAST, ClustalW and HMM) to build (pairwise) alignments, motif searches (SignalP and TMHMM ) and degenerate Position Specific Scoring Matrix (PSSM) searches to identify elements of gene structure [18] including splice sites, branch points, polypyrimidine tracks and translational start sites. A flow diagram of the consecutive steps undertaken in the ABFGP method is presented in Figure 2. Graph-theory is used to translate pairwise alignments of sequences, open reading frames (ORFs), sequence elements or positional attributes to multiple alignments of these entities. The gene similarity graph is an estimation of the gene tree and is used to favor, demote or remove nodes and edges from the ORF similarity graph. Inconsistencies or missing data in series of multiple aligned ORFs trigger a more sensitive HMMER protein search, which can identify missing ORFs of target or informant genes or can recognize lower similarity. The ABFGP method accurately predicts intron-exon boundaries by exploiting ORF (dis)continuity surrounding intron presence-absence patterns [19]. In contrast to *ab initio* gene prediction software, the ABFGP method is able to cope with sequence errors (SEs) and true disruptive mutations (DMs), and recognizes those as inconsistencies in coding region continuity. A quality check on the similarity graph is performed at various stages during ABFGP execution, which can result in removal of an informant once recognized as too distinct. In case the target gene locus is distinct from all informants (which are all homologous to each other), they are all removed. Once the number of informants drops below a user-adjustable threshold (by default set at four), execution of the method is aborted. Finally, an *a posteriori* introspection procedure is applied to each intron and exon in the predicted gene model which assigns a reliability label (‘ok’ or ‘doubtful’) to the predicted gene model.

The output of an ABFGP execution is a GFF file containing the predicted gene model and several features that assist manual inspection of the predicted gene model. Input for ABFGP is a list of orthologous gene loci, of which one is assigned as the target locus to be re-annotated, and all others serve as informants. This resulting list of gene encoding loci is provided as a multi fasta file. A second input option provides additional functionality, where each (informant) gene locus is a folder that contains the genomic locus (fasta format), optionally its currently annotated gene model (gff format) and unigenes aligned to this locus (gff format). A provided unigene is used as an additional informant, from which spliced alignments are exploited as guidance to infer intron-exon boundaries to enhance the prediction performance. A provided gene structure is used to speed up similarity searches by prioritization and to visualize differences between current annotation and the ABFGP prediction. Optionally, the exons of provided genes can be used as prior knowledge to facilitate detection of poorly conserved parts of the gene.

**Figure 3:**
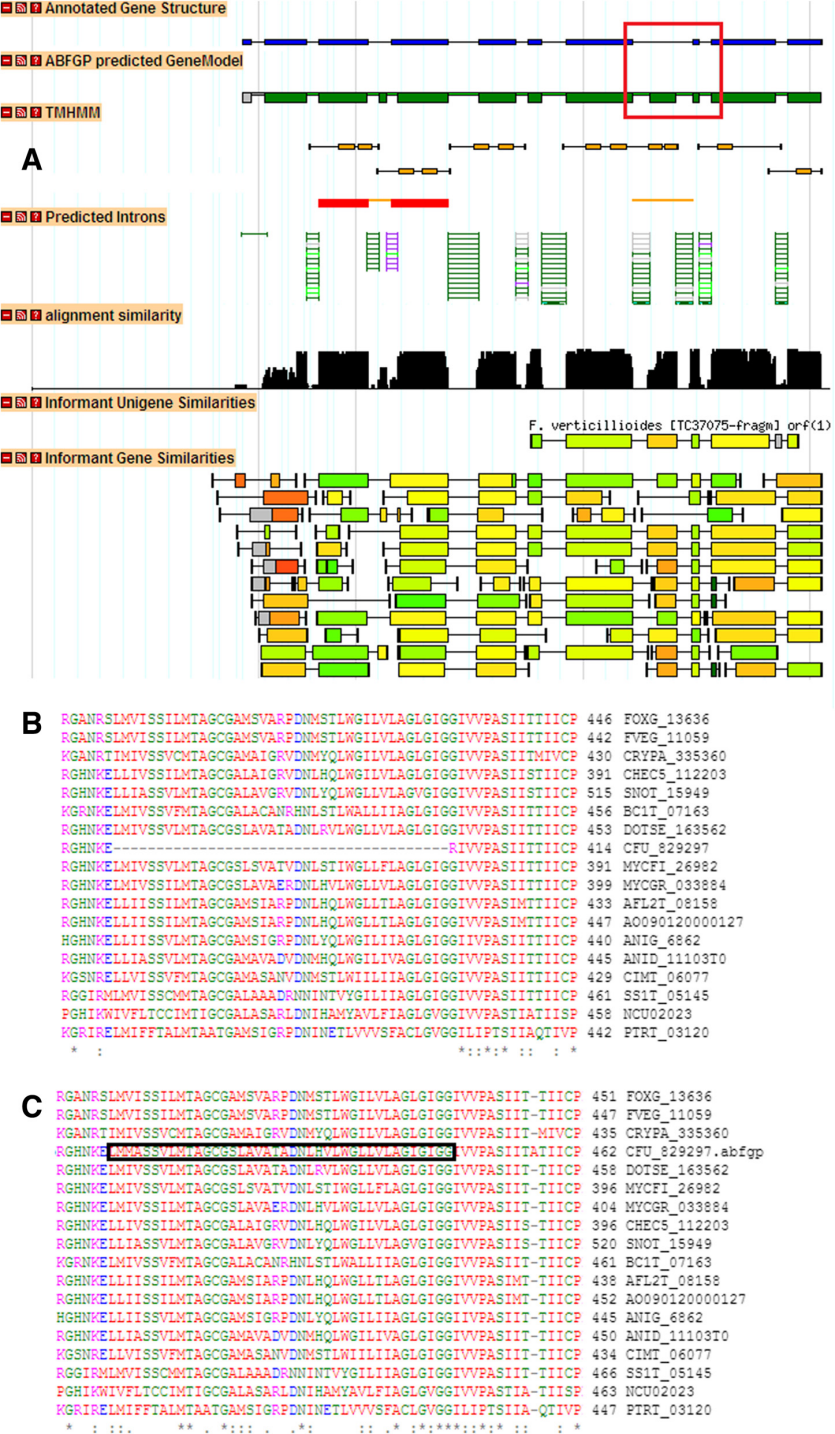
**ABFGP-based curation of the MFS transporter-encoding gene Cf189922 of *Cladosporium fulvum. *A.** Selected tracks of the GFF results obtained by applying ABFGP on the *Cf189922* gene locus using 17 fungal informant genes. The annotated (blue) and the ABFGP-predicted gene model (green and grey) are shown on top. The grey part of the ABFGP prediction indicates an intron-exon boundary with status ‘doubtful’. Below are indicated the introns (orange) and exons (red) that were revised; the red box highlights the site of the second revision. The intron evidence track lists intron-exon boundaries obtained from informants; the colours used in the informant gene similarity track represent a measure for pairwise amino acid similarity. The alignment similarity track represents a summed representation of the inferred multiple sequence alignment of all informants. **B.** Multiple protein sequence alignment of currently annotated gene models of *Cf189922* and its informants. Sequence is restricted to the red box shown in panel A. **C.** Multiple protein sequence alignment of the ABFGP-revised gene model of *Cf189922* and its informants. Sequence is restricted to the red box shown in panel A. The proposed revision is highlighted in the black box.

An example of a re-annotated gene model by ABFGP is given in Figure 3A. It illustrates the predicted gene model at the genomic locus that encodes a Major Facilitator Superfamily (MFS) transporter (Cf189922) in the *Cladosporium fulvum* genome. In this example ABFGP proposes two revisions compared to the originally annotated gene model. Introns (orange) and exons (red) with revised nucleotides are indicated in a separate track. Both revisions involve inclusion of novel exons that split up one intron into two smaller ones. The multiple protein sequence alignment around the second proposed revised site is shown for the unrevised (Figure 3B) and revised (Figure 3C) model. The improved continuity and quality of the sequence alignment suggest that the proposed revision is most likely correct. Moreover, TMHMM prediction performed on a 3-frame translation of the complete locus assigns two trans-membrane helices in the revised exon, which is consistent with the secondary structure of the proteins encoded by the informant gene loci (data not shown). Finally, the additional exon is supported by a partial unigene aligned to the informant gene locus of *Fusarium verticillioides* (TC27075). A more detailed description of the ABFGP method is provided in Additional file 2.

### Benchmarking of the ABFGP method

To benchmark the performance of the ABFGP method, we selected genes from ten different fungi, for which their intron-exon structure is confirmed by full-length unigenes. Of those, 6,965 genes have at least four reliable informant gene loci and passed all selection criteria (Additional file 3; an excel file with all gene identifiers is available at http://tinyurl.com/k9qft5o). Using this dataset, the ABFGP method achieves an overall gene sensitivity of 79.4% (Table 1; Additional file 4), meaning that on average 79 out of 100 gene models are predicted correctly without a single nucleotide error in their overall intron-exon structure.

**Table 1:** Benchmarking of the ABFGP performance on validated genes compared to GeneMark-ES

**Species**		**10 pooled species**^**1**^	***Magnaporthe oryzae***^***2***^		***Fusarium verticillioides***	
Method		ABFGP	ABFGP	GeneMark-ES	ABFGP	GeneMark-ES
# unigenes		6,965	956	169	1154	327
Intron	Sn	91.16	91.5	89.3	92.2	90.7
	Pr	97.08	97.4	90.5	98.2	94.3
Exon	Sn	88.54	89.1	88.0	90.4	85.4
	Pr	98.91	99.4	89.1	98.9	87.9
Nucleotide	Sn	98.75	98.3	98.2	99.3	98.8
	Pr	99.08	99.3	97.1	99.0	97.1
Gene^3^	Sn	79.4 (5,533)	81.7 (781)	n.a.	82.1 (947)	n.a.

Sensitivity (Sn) and precision (Pr) of the gene model components (introns, exons, nucleotides) are expressed in percentages. Sn is calculated as true positives divided by: (true positives + false negatives); and Pr as true positives divide by: (true positives + false positives) [3].

^1^A list of all ten fungal species and results per species are provided in Additional file 4.

^2^Formerly named *Magnaporthe grisea.*

^3^The gene sensitivity is the percentage of gene models that is predicted without a single error. Total number of correctly predicted gene models is indicated in between brackets. Gene sensitivity was not provided for GeneMark-ES.

The ABFGP method applied to a set of available full-length unigenes from *Magnaporthe oryzae* and *Fusarium verticillioides* was compared to GeneMark-ES [5], which was previously used on a smaller set of unigenes from these two fungi (Table 1). The ABFGP method performed better than GeneMark-ES on all gene components (exons, introns and nucleotides), in terms of sensitivity but most noticeably in terms of precision. The gene sensitivity achieved by ABFGP was 81.7% and 82.1% for the unigenes of *M. oryzae* and *F. verticilloides*, respectively. The results of this benchmarking show that the ABFGP method can confidently be applied to improve gene models in fungal genomes.

### ABFGP as a tool to curate gene models of six annotated fungal genomes

To illustrate its versatility, we applied the ABFGP method on the gene catalogue of six different fungal species previously sequenced and annotated at the BROAD [2] and JGI institutes [1]: *Botrytis cinerea*, *Cladosporium fulvum, Dothistroma septosporum, Mycosphaerella fijiensis, Verticillium dahliae* and *Zymoseptoria tritici* (Table 2). ABFGP was performed after selection of eligible genes (BDBH category) based on informant genes retrieved from a set of 29 fungal genomes (Additional file 1). A second, much smaller set of genes was compiled from informants that suggested species-specific variation or gene models with errors (GME). Between 7,285 and 8,504 annotated gene loci per species were eligible for ABFGP using these criteria. For 0.4-2.0% of these, ABFGP was aborted during execution because the number of representative informants dropped below four during the integrated quality assessment. For the remaining loci, the gene models predicted by ABFGP were compared to their current annotations. As expected, the currently available annotation and the ABFGP-predicted structures of a large fraction of the gene models (on average 68%) were identical (Table 2). Predicting the same intron-exon-structure by two independent methods is a strong indication that the predicted gene models are correct. The ABFGP method proposed at least a minor revision for 22% to 41% of the assessed genes in a given species. Among those, the GME category of genes is highly overrepresented, with 62% to 75% of them being revised versus only 19% to 34% from the BDBH category. The lowest number of revisions is proposed for the most recently annotated genomes of the fungi *C. fulvum* and *D. septosporum*[20], and the highest number for *B. cinerea*. This is likely due to the fact that the genome assembly of *B. cinerea* has a low sequence coverage produced by Sanger technology, and its annotation was performed by older, less accurate gene predictor software (Table 2).

**Table 2:** Gene models in six fungal species re-annotated by the ABFGP method

**Species**	***Botrytis cinerea***		***Cladosporium fulvum***		***Dothistroma septosporum***		***Mycosphaerella fijiensis***		***Verticillium dahliae***		***Zymoseptoria tritici ***^**1**^	
Sequence technology	Sanger		454		Illumina/454/Sanger		Sanger		Sanger		Sanger	
Fold genome coverage	4.5		21		34		7.1		7.5		8.9	
# Annotated genes	16,448		14,127		12,580		10,313		10,535		10,952	
Annotation pipeline^2^	BROAD		GeneMark-ES		JGI		JGI		BROAD		JGI	
Annotation year^3^	2005		2009		2010		≤2008		2008		2008	
Reference	[21]		[20]		[20]		n.a.		[22]		[23]	
Total eligible gene models	8,503		7,574		8,090		7,283		8,362		7,893	
Bi Directional Best Hit	7,165		6,990		7,511		6,773		7,814		7,317	
Gene Model Error	1,338		584		579		510		548		576	
Confirmed/unchanged^4^	4,832	57%	5,823	77%	6,249	77%	4,775	66%	5,390	64%	5,262	67%
Revised^4^	3,505	41%	1,724	23%	1,770	22%	2,456	34%	2,870	34%	2,553	32%
Bi Directional Best Hit^5^	2,481	35%	1,304	19%	1,404	19%	2,064	30%	2,511	32%	2,137	29%
Gene Model Error^5^	1,024	77%	420	72%	366	63%	392	77%	359	66%	416	72%
Aborted^4^	166	2,0%	27	0,4%	71	0,9%	52	0,7%	102	1,2%	78	1,0%

^1^Formerly named *Mycosphaerella graminicola*.

^2^Sequencing centre which sequenced and annotated this genome (BROAD institute or Joint Genome Institute); *C. fulvum* was sequenced at Wageningen University and annotated using GeneMark-ES version 2.2 [20].

^3^Estimated year the gene calling was performed.

^4^Number and percentage of all gene models in this category.

^5^Number and percentage of revised gene models in this category.

### Reliability of the ABFGP-predicted gene models

The ABFGP method confirmed 57 to 77% of the previously reported annotated gene models and proposed revisions for the remaining in six fungal species (Table 2). The overall quality of the revised predictions is supported by high accuracy as shown by the benchmarking on unigenes (Table 1). To address the reliability at the level of individual genes, the ABFGP method was equipped with an *a posteriori* introspection module. Each intron and exon in the multiple gene model alignment was evaluated on a series of stringent criteria (e.g. alignment quality, length variance, splice site score, etc.) and was labelled to indicate the likelihood of its correctness: gene models were labelled as ‘ok’ only if all individual introns and exons received this label, and were labelled as ‘doubtful’ in case one or more introns or exons received this label (Table 3). Of the confirmed gene models on average 86.4% was labelled ‘ok’ and 13.6% ‘doubtful’ , whereas of the revised gene models 66.1% was labelled ‘ok’ and 33.9% ‘doubtful’. The introspection procedure was also applied to the benchmark set of 6,965 genes supported by unigenes and resulted in 5,016 true positives (72.2%), 496 true negatives (7.1%), 533 false negatives (7.7%) and 899 false positives (12.9%). This analysis shows that the introspection procedure is quite accurate, and that the majority of ABFGP-revised models of the re-annotated genomes is reliable.

**Table 3:** Introspection of results obtained by the ABFGP method

**Species**	***Botrytis cinerea***		***Cladosporium fulvum***		***Dothistroma septosporum***		***Mycosphaerella fijiensis***		***Verticillium dahliae***		***Zymoseptoria tritici***^**1**^		**Pooled unigenes**^**2**^
Total number of assessed genes^3^	8,337		7,547		8,019		7,231		8,260		7,815		6,965
Confirmed/unchanged	4,832		5,823		6,249		4,775		5,390		5,262		Correct
Labeled ‘ok’^4^	3,942	82%	5,186	89%	5,505	88%	4,216	88%	4,536	84%	4,539	84%	5,015 (TP)
Labeled ‘doubtful’^4^	890	16%	637	11%	744	12%	559	12%	854	16%	723	16%	533 (FN)
Revised	3,505		1,724		1,770		2,456		2,870		2,553		Incorrect
Labeled ‘ok’^4^	2,137	61%	1,160	67%	1,209	68%	1,730	70%	1,864	65%	1,734	68%	899 (FP)
Labeled ‘doubtful’^4^	1,368	29%	564	33%	561	32%	726	30%	1,006	35%	819	32%	496 (TN)

^1^Formerly named *Mycosphaerella graminicola.*

^2^Correctly predicted gene models (benchmarked on the full-length unigenes) that were labelled by the introspection procedure as ‘ok’ are true positives (TP) and labelled ‘doubtful’ are false negatives (FN). Genes that were incorrectly predicted and were labelled ‘ok’ are false positives (FP) and labelled ‘doubtful’ are true negatives (TN).

^3^Total eligible number of genes minus number of genes aborted during execution (Table 2).

^4^Number and percentage of genes that are labelled ‘ok’ and ‘doubtful’ by the introspection procedure in each category.

### Types of revisions proposed by the ABFGP method

The most conspicuous differences between the annotated and ABFGP-predicted gene models are summarized in Table 4. Major revisions proposed by the ABFGP method comprise corrections of falsely fused and split gene models in current annotations. *B. cinerea* appears enriched for both incorrectly merged and split genes and *C. fulvum* for incorrectly merged genes. Up to 19% of the revisions proposed by the ABFGP method are due to SEs and/or DMs, which were particularly often encountered in genes of *B. cinerea*, *C. fulvum* and *V. dahliae*. Other revisions involve boundary changes, removal and addition of exons and introns in predicted gene models. Additional exons are more rarely predicted, but they are frequently occurring as internal revisions (as shown in Figure 3 for *C. fulvum*) of genes in *B. cinerea* and *V. dahliae*. ABFGP frequently removed stopless 3n introns in the gene models of *M. fijiensis* and *Z. tritici*. The proposed revisions resulted mainly in a decrease of the average intron length: -42, -35, -30, -29, -6 and +1 nucleotides for *M. fijiensis, B. cinerea*, *C. fulvum, Z. tritici, V. dahliae* and *D. septosporum*, respectively.

**Table 4:** Types of revisions in annotated gene models made by the ABFGP method

**Species**	***Botrytis cinerea***		***Cladosporium fulvum***		***Dothistroma septosporum***		***Mycosphaerella fijiensis***		***Verticillium dahliae***		***Zymoseptoria tritici***^**1**^	
Total revised genes^2^	3,473		1,721		1,761		2,448		2,865		2,552	
Genes containing SE and/or DMs^3^	353		333		176		127		515		66	
Genes split by ABFGP	195		183		62		91		94		130	
Genes merged by ABFGP	102		12		16		27		19		28	
Total annotated exons	12967		5675		5211		7525		10709		8316	
Unrevised	5970		2372		2078		2593		4956		3116	
Boundary revision^4^	4851		2274		2341		3230		4355		3357	
5’ or 3’ removed (−) / added (+)^5,6^	−783	+617	−451	+252	−265	+224	−297	+341	−529	+333	−415	+335
Internal removed (−) / added (+)^5,7^	−51	+616	−20	+98	−24	+35	−59	+74	−66	+346	−76	+75
Total annotated introns	9459		3947		3438		5058		7838		5753	
Unrevised	4907		2019		1740		2276		4048		2836	
Boundary revision^8^	1799		692		727		889		1738		839	
Stopless 3n removed (−) / added (+)^9^	−447	+189	−166	+146	−365	+146	−1032	+99	−331	+244	−953	+130

^1^Formerly named *Mycosphaerella graminicola.*

^2^The total number of revisions can exceed the total number of revised genes because a gene model can contain more than one revision.

^3^Genes for which the revision(s) include sequence errors or mutations.

^4^Exons with a different start and/or end coordinate when comparing both gene models.

^5^Exons incorporated in only one of both gene models (not in the ABFGP model/only in the ABFGP model).

^6^Omitted and additional exons in recognized false gene splits and fusions were not counted.

^7^(Large) intron in one gene model, split into two smaller introns with intermediary (small) exon in the other gene model.

^8^Introns with a different donor and/or acceptor site when comparing both gene models.

^9^Stopless 3n introns incorporated in only one of both gene models (not in the ABFGP model/only in the ABFGP model).

### Increasing the number of informants improves performance of the ABFGP method

ABFGP performance decreased when using fewer informants or when closely related informants are not available (data not shown). For the curation of a particular gene model, the most closely related fungal species failed to provide informants for 7 to 19% of selected loci (Additional file 5). Conversely, fungal species that provided the lowest number of informants still contributed 16 to 38% of informant loci. In addition, in some cases, fungal species that provided most of the informant loci are not always the closest relatives. For example, *M. fijiensis*, the closest relative of *Z. tritici*, is not among the top three species that provided the highest number of informants (Additional file 5). Similarly, *N. haematococca* and *M. oryzae* provide more informants than *V. albo-atrum* for the curation of *V. dahliae*. For *C. fulvum* and *M. fijiensis*, it is striking that fungi that belong to a different taxonomic class are in the top three species that provided the highest number of informants. Our results show that the six studied fungal gene catalogues differ in quality. Because all informant catalogues were predicted by the same genome sequence centres (see Additional file 1), similar error rates are expected to occur in their gene models. An unexpected low contributor to the pool of informants could be explained by a slightly higher error rate in its gene catalogue. In addition, many genes show a discontinuous distribution in the fungal tree of life [8,10]. This underlines the importance of selecting informants from a wide phylogenetic spectrum of species rather than from a small set of closely related species.

## Discussion

### The ABFGP method accurately predicts intron-exon structures of protein-encoding genes in fungi

The ABFGP method can accurately re-annotate the intron-exon structure in a gene-by-gene fashion when a gene locus is provided with sufficient informants. GeneMark-ES was chosen as a state of the art *ab initio* gene predictor, and we have shown that the ABFGP method improves the quality of the gene models. This is explained by a higher precision (Table 1), which means that a lower number of false positives are reported by ABFGP. Indeed, in general, evidence- or alignment-based methods are less prone to wrongly assign additional exons [3], because they are only predicted when supported by informants. Predicting introns in compact genomes with numerous small introns is challenging [5], yet ABFGP achieves both a high sensitivity (91.2%) and precision (97.3%) (Table 1). This is achieved by exploiting abundantly occurring intron presence-absence patterns [19]. SEs and/or DMs can be confidentially recognized as discontinuities when compared with exonic sequences of informant genes. Finally, lack of synteny in distantly related fungi facilitates recognition of false gene fusions, which is a frequently observed error made by *ab initio* gene predictors [5,16]. Adjacent genes with the same orientation are prone to be falsely fused to the target gene, but this is minimized in the ABFGP method because of the shuffled gene order in informant genomes. Whole-genome alignment-based gene prediction benchmarked on a test set of 1,483 genes from two strains of *C. neoformans* achieved 88% and 89% exon sensitivity and precision, respectively, resulting in an overall gene sensitivity of ~60% [6], which is low considering the high conservation between the two genomes. This shows that the gene-by-gene approach by the ABFGP method is more powerful, even by making use of informant genes from evolutionary distant fungal species. The benchmark test showed uniform performance on unigenes from ten selected species (Additional file 4). Yet, this performance was, in case of *D. septosporum,* achieved with generic PSSMs that were not derived from its own splice sites. Species-specific parameterization of gene properties was indicated as crucial for the performance of *ab initio* supervised [4], unsupervised [5] as well as the alignment-based gene prediction methods [6]. We speculate that in the ABFGP method, the number of informants compensates for the absence of species-specific parameterization.

### ABFGP as a genome-wide annotation assessment tool

Between 7,205 and 8,270 gene models of six fungal genomes were automatically assessed by the ABFGP method. Between 1,724 and 3,505 (on average 2,480) of these gene models were proposed to be incorrect and needed revision. A more stringent indication of correct revisions is obtained by counting only those revised gene models that were labelled ‘ok’ (Table 2), corrected for the observed error rate of the ABFGP method (based on 79% gene sensitivity). This yields an estimated revision of between 1,362 and 2,769 gene models for each fungal species. These numbers are in the same range as those obtained in a recent genome-wide re-annotation effort of the *F. graminearum* genome, which was based on predictions by a suite of gene predictors, using expression data and followed by extensive manual curation [16]. In that case, 1,770 gene models were revised, 691 new gene models were added and 286 gene models were removed. Yet, a recent study using RNA-Seq data revised another 655 gene models [17], showing that the quality-improving manual curation effort was not yet exhaustive. Their analysis [16] and ours independently show that thousands of genes are still wrongly annotated in gene catalogues of many published fungal genomes. Interestingly, the same types of revision were reported (false gene splits and fusions, novel introns and a decrease in average intron length) as those proposed by the ABFGP method.

Types of revision are often related to the annotation pipelines used (Table 2). For example, inclusion of new exons represents a rare class of revisions, except in the two genomes that were annotated at the BROAD institute. In contrast, prediction of too many stopless 3n introns was observed in the genomes of *M. fijiensis* and *Z. tritici* that were sequenced at the JGI. The lowest number of revised gene models was proposed for *C. fulvum* and *D. septosporum*, which represent the most recently sequenced and independently annotated genomes [20]. We speculate that this might reflect the steady increase in accuracy of *ab initio* gene prediction software.

In this study six different fungi from three distinct phylogenetic classes were re-annotated, using informants from five classes of Ascomycota and two unrelated Basidiomycota. This shows that the ABFGP method is species-independent and can be applied to a wide variety of fungal genomes.

Genome-wide re-annotation by the ABFGP method did not capture the complete gene catalogues (Table 2) which is mainly due to the stringent criteria that were chosen to obtain the most likely orthologous informant genes (see Methods). This effect is most obvious for informant genes obtained from poorly annotated genomes. Performance for those genes can be improved, besides lowering this threshold, by expanding beyond using annotated genes only. An informant locus can be any genomic region that has ample sequence similarity to the target protein or locus. TBLASTN or TBLASTX could be used to detect loci that failed to be recognized and annotated as protein-coding genes or were poorly annotated (see Figure 1). Loci that are obtained directly from a (non-annotated) genomic sequence could be used as an additional resource for informants that would simultaneously increase the number of eligible target genes and prediction performance of ABFGP. The reverse strategy could also be employed by using the ABFGP method to generate *de novo* gene models in the target genome that lack predicted gene models but have significant sequence similarity to predicted proteins in other species. However, a general limitation of *de novo* evidence-based gene prediction, including the ABGFP method, is that annotation of species-specific or fast evolving genes is not possible by any prediction method. The ABFGP method follows an alternative approach to the various other *ensemble* predictors, because it derives its evidence directly from genomic informant sequences. Moreover, it proposes revised gene models that include SEs and/or DMs. This makes the ABFGP method complementary to other ensemble predictors, because these occur frequently in the gene catalogues of these fungal genomes [24].

### Sequence errors and disruptive mutations in fungal genes

Presumed inconsistent gene models were revised in 70 to 83% of all cases (Table 2), of which on average 55% were labelled by the introspection procedure as ‘ok’ for all introns and exons. Among these revisions was an unexpected high number of gene models containing SEs and/or DMs. Because *ab initio* gene prediction software does not allow in-frame stops or frame-shifts causing indels, (pseudo)genic regions with strong coding signals will often be predicted to be truncated or split gene model(s). Of the six studied fungi, most revisions were proposed for *B. cinerea*, likely because its Sanger sequenced genome assembly is supported by 4.5× coverage only [21], and its annotation was performed several years ago. Recently, resequencing of *B. cinerea* using Illumina, supplemented with some additional small Sanger reads, resulted in a new assembly with 50× coverage [25]. This new sequence not only revealed 31,275 SEs (personal communication Dr. Martijn Staats), but also a considerable number of assembly errors in the original reference sequence, of which many were located in coding regions that contained annotated, yet apparently fragmented genes (personal communication Dr. Jan van Kan). This could be an explanation for the higher frequency (2.0% versus 0.4-1.2% for the other five fungi, Table 2) of abandoned executions by the ABFGP method. However, a considerable fraction of inconsistencies observed in coding regions were confirmed by resequencing, indicating that they were not SEs but true DMs. Additional studies on DMs in these six fungal species suggest that pseudogenization is very common in fungi [24]. Our results show that many fungal gene catalogues still contain numerous unidentified truncated and erroneous gene models due to SEs and/or DMs, that are readily detected by the ABFGP method.

### Introspection of proposed gene model revisions

The introspection module for assessing gene model correctness is a useful extension of the ABFGP method as it helps to prioritize gene models that still need manual curation. For the six fungal genomes, between 3,942 and 5,505 genes were suggested to not require additional manual curation (Table 3). Based on the benchmarked performance of the introspection procedure using the unigene dataset, the error rate of genes incorrectly labelled as ‘ok’ is estimated to be 12.9%. This accounts for only 500 to 700 models out of 4,000 to 5,500 that contain errors. For gene models that were recognized as ‘doubtful’, the ABFGP method provides a GFF-track that shows the doubtful parts of the predicted gene model that require manual curation. However, the introspection module still needs further improvement because 20.6% of the gene models is incorrectly labelled: 12.9% is labelled as ‘ok’ but do contain (small) errors and 7.7% is labelled as ‘doubtful’ whereas the gene models are correct. Lowering the number of false positives can possibly be achieved by including *ab initio* gene model prediction in the ABFGP method, which would allow better detection of species-specific variation of genic regions. This would further increase the efficiency of the ABFGP method as an automated and accurate method for gene model curation.

## Conclusions

Availability of an accurate gene catalogue of an organism is a prerequisite and starting point for functional analyses of its genes. Obtaining such a catalogue with minimal manual input is still a major challenge. The ABFGP method is a useful tool to integrate into existing gene annotation pipelines because it can assess and improve gene models with great accuracy in a fully automated manner. The concept of gene-by-gene alignment-based gene prediction exploits the availability of dozens of sequenced fungal genomes, which is particularly useful for annotating novel genomes of these plastic organisms. The possibility of the ABFGP introspection procedure at the gene and intron-exon level helps to decrease the number of gene models that still require manual curation. Because fungal genome sequencing is undertaken at an accelerating pace [1], both quality and number of informant gene loci are expected to increase in the coming years, which will disclose more target gene loci in genomes and also increase the efficiency and reliability of the ABFGP method.

## Methods

### Sequences, annotations and third party software used

Genomes, proteomes and annotations of 29 fungal species were downloaded from the Fungal Genome Initiative of the BROAD Institute [2] and the Fungal Genomics Program of the Joint Genome Institute (JGI) [1] (Additional file 1). Available unigenes from ten fungal species were downloaded from the JGI and The Gene Index Project (http://compbio.dfci.harvard.edu/tgi/). The ABFGP method uses several third party applications: BLAST 2.2.8, ClustalW 2.0.12, HMMER 2.3.2, SignalP 3.0, TMHMM 2.0, transeq, getorf and tcode from EMBOSS 6.2.0.

### Full-length unigenes

Datasets of assembled unigenes (Additional file 1) were aligned to their genomes using GeneSeqer (October 2005) and for each unigene the obtained intron-exon structure of its coding sequence was compared to its annotated gene model. For benchmarking the ABFGP method only those unigenes that were full-length were selected.

### Informant selection

An all-versus-all similarity matrix was created between all proteins from the 29 predicted proteomes using BLASTP. From this matrix, informant proteins from different fungi were selected for each target protein by applying the following criteria: the protein must represent (i) the bi-directional best hit (BDBH) in the informant’s proteome, (ii) the alignment must span at least 70% of the length of both target and informant protein, (iii) the relative difference in length between target and informant protein must be below 50% (calculated from ii) and (iv) the alignment‘s bitscore between target and informant protein must be at least 10% of the bitscore of the proteins when compared to themselves. As a final criterion, at least four informant proteins must be available for a target protein, and the total number of informants was limited to the 19 most similar informants (based on bitscore). This dataset of genes eligible for ABFGP is referred to as BDBH. A second category was created by lowering the requirement of length coverage to 25% and increasing length difference to 300%, followed by filtering for target proteins that were linked to either consistently longer or shorter informant proteins. Consistent protein length variation putatively indicates species-specific variation or that the corresponding gene model contains major errors (this dataset is referred to as GME). For both categories, target and informant proteins were loaded into ABFGP as DNA sequence of their genomic locus flanked by an additional 1.5 kb of sequence on both sides of the gene’s start and stop codon. Unigenes aligned to these gene loci were taken along as additional informants. In the benchmark that uses unigenes, informants were selected only by the BDBH approach and full-length unigene data aligned to the target gene locus were discarded; the parameters `--abinitio` and `--benchmark` were used to discard the unigene of the target locus and annotated gene models as hints. In all benchmark analyses, sensitivity and precision are calculated according as described by Picardi and Pesole [3], in which specificity is an alias for precision.

### Position Specific Scoring Matrices of genic elements

Definitions of donor site, acceptor site, branch point and polypyrimidine tracks were chosen according to [18]. Generic fungal PSSMs (Additional file 2) for the canonical donor (n = 571,185), the non-canonical GC donor (n = 2,428) and the canonical acceptor (n = 576,021) were derived from all splice sites without any nonambiguous nucleotide in 25 annotated genomes (excluding the annotations of *Cladosporium fulvum*, *Coccidioides posadasii*, *Dothistroma septosporum*, *Nectria haematococca* and *Trichoderma atroviride*, which were added as target and/or informant species in a later stage of the analyses).

### Access to the method and data

A technical explanation of the ABFGP method, and its GFF visualization is provided in Additional file 2. The source code of the ABFGP method is available (see Availability and requirements). Other datasets are available upon request by the corresponding authors: the complete list of unigene identifiers used for the benchmark analyses (.xls), the predicted gene models from the benchmark that uses unigenes (GFF files) and the genome-wide re-annotation of the six fungi (fasta and simplified GFF files).

## Availability and requirements

**Project name:** ABFGP

**Project home page:**https://github.com/atevanderburgt/ABFGP

**Operating system:** Linux, Unix

**Programming language:** Python

**Other requirements:** Python 2.6 or higher

**Licence:** GNU GPL

Any restrictions to use by non-academics: None

## Competing interests

The authors declare that they have no competing interests.

## Authors’ contributions

AVDB devised the method and created the tool in consultation with ES and PDW. AVDB implemented it on the selected dataset of fungi. AVDB, JC and PDW wrote the manuscript. All authors read and approved the final version of manuscript.

## Supplementary Material

Additional file 1 **Fungal genomes used for alignment-based fungal gene prediction.** Fungal genomes and their phylogeny used in this study. Click here for file

Additional file 2 **Explanation of the ABFGP method.** In-depth explanation of the ABFGP method. Click here for file

Additional file 3 **Determination of a dataset from ten fungi for benchmarking the ABFGP method.** Determination of a dataset of 6,965 experimentally validated genes models from ten fungal genomes for benchmarking the performance of the ABFGP method. Click here for file

Additional file 4 **Benchmarking results of ABFGP performance.** Benchmarking results of ABFGP performance on 6,965 experimentally validated gene models from ten fungal species. Click here for file

Additional file 5 **Rank of species providing informant gene loci used for the six re-annotated gene catalogues.** Top three and bottom two species that provided the highest number of informants for the re-annotation of the gene catalogues of six fungal species. Click here for file
