# Supplementary material for: Automated alignment-based curation of gene models in filamentous fungi
Source: BMC Bioinformatics. 2014 Jan 16;15:19. doi: 10.1186/1471-2105-15-19 (PMC3898260; doi:10.1186/1471-2105-15-19)
Supplement: Additional file 1 — Fungal genomes used for alignment-based fungal gene prediction. Fungal genomes and their phylogeny used in this study. [file 1471-2105-15-19-S1.doc]

**Additional File 1: fungal genomes used for alignment based fungal gene prediction.**

For each species is given the classification according to the NCBI Taxonomy browser (12/06/2012). The source indicates the sequence repository from which the genome data were downloaded. Species from which unigenes were used to benchmark the performance of the ABDGP method are indicated

| **Fungal species** | **Phylum | Class | Order** | **Source^a^** | **Unigenes^b^** |
| --- | --- | --- | --- |
| *Aspergillus flavus* | Ascomycota | Eurotiomycetes | Eurotiales | BROAD |  |
| *Aspergillus nidulans* | Ascomycota | Eurotiomycetes | Eurotiales | BROAD | GI5 |
| *Aspergillus niger* | Ascomycota | Eurotiomycetes | Eurotiales | BROAD |  |
| *Aspergillus oryzae* | Ascomycota | Eurotiomycetes | Eurotiales | BROAD |  |
| *Botrytis cinerea* | Ascomycota | Leotiomycetes | Helotiales | BROAD |  |
| *Candida albicans* | Ascomycota | Saccharomycetes | Saccharomycetales Saccharomycetales | BROAD |  |
| *Cladosporium fulvum* | Ascomycota | Dothideomycetes | Capnodiales | JGI |  |
| *Cochliobolus heterostrophus* C5 | Ascomycota | Dothideomycetes | Pleosporales | JGI | JGI |
| *Coccidioides immitis* RS | Ascomycota | Eurotiomycetes | Onygenales | BROAD |  |
| *Cryptococcus neoformans grubii* h99 | Basidiomycota | Tremellomycetes | Tremellales | BROAD |  |
| *Coccidioides posadasii* | Ascomycota | Eurotiomycetes | Onygenales | BROAD |  |
| *Cryphonectria parasitica* | Ascomycota | Sordariomycetes | Diaporthales | JGI | JGI |
| *Dothistroma septosporum* | Ascomycota | Dothideomycetes | Capnodiales | JGI | JGI |
| *Fusarium graminearum* | Ascomycota | Sordariomycetes | Hypocreales | BROAD |  |
| *Fusarium oxysporum* | Ascomycota | Sordariomycetes | Hypocreales | BROAD |  |
| *Fusarium verticillioides* | Ascomycota | Sordariomycetes | Hypocreales | BROAD | GI8 |
| *Histoplasma capsulatum* NAm1 | Ascomycota | Eurotiomycetes | Onygenales | BROAD |  |
| *Magnaporthe oryzae* ^c^ | Ascomycota | Sordariomycetes | Magnaporthales | BROAD | GI6 |
| *Mycosphaerella fijiensis* | Ascomycota | Dothideomycetes | Capnodiales | JGI |  |
| *Neurospora crassa* | Ascomycota | Sordariomycetes | Sordariales | BROAD | GI4 |
| *Nectria haematococca* | Ascomycota | Sordariomycetes | Hypocreales | JGI | JGI |
| *Puccinia graminis tritici* | Basidiomycota | Pucciniomycetes | Pucciniales | BROAD |  |
| *Pyrenophora tritici-repentis* | Ascomycota | Dothideomycetes | Pleosporales | BROAD |  |
| *Stagonospora nodorum* | Ascomycota | Dothideomycetes | Pleosporales | BROAD |  |
| *Sclerotinia sclerotiorum* | Ascomycota | Leotiomycetes | Helotiales | BROAD |  |
| *Trichoderma atroviride* | Ascomycota | Sordariomycetes | Hypocreales | JGI | JGI |
| *Verticillium dahliae* VdLs.17 | Ascomycota | Sordariomycetes | Glomerellales | BROAD |  |
| *Verticillium albo-atrum* VaMs.102 | Ascomycota | Sordariomycetes | Glomerellales | BROAD |  |
| *Zymoseptoria tritici* ^d^ | Ascomycota | Dothideomycetes | Capnodiales | JGI | JGI |

^a^ BROAD institute (www.broadinstitute.org) and Joint Genome Institute (www.jgi.doe.gov)

^b^ The Gene Index Project, including version number (compbio.dfci.harvard.edu/tgi/) and Joint Genome Institute ([www.jgi.doe.gov](http://www.jgi.doe.gov/))

^c^ Formerly named *Magnaporthe grisea*

^d^ Formerly named *Mycosphaerella graminicola*
