# Supplementary material for: Automated alignment-based curation of gene models in filamentous fungi
Source: BMC Bioinformatics. 2014 Jan 16;15:19. doi: 10.1186/1471-2105-15-19 (PMC3898260; doi:10.1186/1471-2105-15-19)
Supplement: Additional file 2 — Explanation of the ABFGP method. In-depth explanation of the ABFGP method. [file 1471-2105-15-19-S2.doc]

**Additional File 2: Explanation of the ABFGP method.**

**Mathematical representation of gene structure alignment**

A DNA sequence *s_0_*, which contains a protein-coding gene, was present in a common ancestor. Before and after several speciation events, the sequence has undergone a series of mutations, gene duplications and intron gain and loss events, resulting in a set of *S* sequences (*s_1_, s_2_, … s_S_*), encoding orthologs or paralogs of a single gene G (*g_1_, g_2_, … g_S_*). Each gene is composed of a number of exons *E_g_* (*e_g,1_, e_g_,_2_, … e_g,x_*), where x can be (in an Ascomycete fungal species) any positive number ranging from one to ten with few exceptions with more exons. By definition, each exon *e* is encoded on an Open Reading Frame (ORF) O (o*_g,1_, o_g_,_2_, … o_g,y_*), where y ≤ x (more than one exon can be encoded on a single ORF).


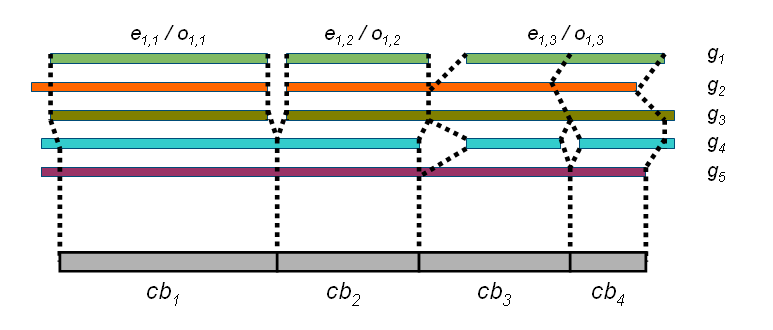


***Figure 1: Schematic representation of a complete gene structure alignment. This gene structure alignment of five genes consists of four coding block graphs (CBGs)***

An example of a (sequence based) gene structure alignment of *S*=5 gene loci is shown in Figure 1. Here, the concept of a coding block is introduced, signifying an aligned block that is in none of the sequences in S interrupted by an intron. A coding block can be represented as a *p,q* graph with *p (=S)* nodes and *q* edges. The nodes are the aligned exon parts of the ORFs (o*_g1,a_, o_g2_,_b_, … o_gS,c_*) and the edges are the pairwise sequence similarity scores between the nodes. The coding block graph (CBG) has *p=S* nodes and *q=S(S-1)/2* edges and is by definition a complete graph *K_S_*. A gene structure multiple alignment can be described by a linearly constrained series of CBGs (*cbg_1_, cbg_2_, … cbg_z_*) where *z* ≥ max(*e_g1_, e_g2_, … e_gS_)*, the largest number of exons *E_g_* in a single gene in *G*. In case of intron presence-absence patterns (PAP), as shown in Figure 1, *z* can be larger than the maximum number of exons in a single gene.

The multiple alignment of a CBG is by definition spatially confined to the extrapolated intersection that is covered by all pairwise alignments. CBGs that share a single pairwise similarity segregate this alignment in distinct ranges for each CBG. Dashed lines in Figure 2 indicate the extrapolated intersections of the CBG and the alignment between the first exon of both *g_4_* and *g_5_* is an example of an disintegrated alignment caused by occurrence of the same exon in both *cbg_1_* and *cbg_2_*. The total weight of a CBG is defined as the sum of the bitscore of the extrapolated confined parts of all pairwise alignments. As a consequence, longer and more similar CBGs give rise to higher scores.

The pairwise alignment information captured in a CBG can be used to estimate a gene tree. In graph representation, this Gene Tree Graph (GTG) defines similarity between nodes (ORFs representing a gene or species), where similarity is expressed as an identity score percentage. The identity score is obtained by scoring identical and similar amino acids as 1 and 0.5, respectively, divided by the length of the alignment. The relative contribution of an edge or a node (and all its edges) to the GTG is obtained by normalizing the identity scores by the GTG’s total weight.

**The core of the ABFGP method**

The ABFGP algorithm aims to reconstruct the graph representation of a gene structure as described above. It does so by creating a Pairwise aligned coding block Collection Graph (PAG) of all ORFs (nodes) that share similarity (edges) with ORFs of all other sequences in S. *K_S_* subgraphs are identified in the PAG and abstracted as an ordered series of CBGs. Next, the CBGs are amalgamated into a gene structure by constructing splice site graphs on the boundaries of the CBGs. In analogy with the CBGs in the PAG, the highest scoring *K_S_* subgraphs in the splice site graphs are the donor and acceptor sites that circumscribe the exons and establish the gene structure. Graph theory is applied as well to determine start and stop location of the gene.

Alongside with the specification of the method, several known common characteristics of genes and of their intron-exon structures are used as prior assumptions and postulated as theorems. Some of these are *a priori* known to be incorrect assumption in a minority of cases only. The validity of these theorems are examined at the very end of the ABFGP method to assess the correctness of its own prediction by introspection.

The PAG is created by translating the set of *S* input DNA sequences (*s_1_, s_2_, … s_S_*) into *F* sets of ORFs in all three reading frames (*f_1_, f_2_, … f_F==S_*), so each ORF set *f_x_* consists of O ORFs (*o_x,1_, o_x,2_ … o_x,O_*) where O depends on sequence length and characteristics of *s_x_*. Orfs are defined from stop to stop of at least 15nt length with EMBOSS getorf. Next, *P* pairwise comparisons *p_xy_* of ORF sets *f_x_* and *f_y_* of distinct input sequences (x≠y, so *P=S(S-1)/2*) are performed with blastp, using a low detection threshold (BLOSUM62, high-scoring segment pairs (HSPs) of at least 12aa length and scoring 40 bits). Starting with the highest (bit)scoring HSP in each pairwise comparison *p_xy_* as an anchor, a scaffolding process is undertaken. Because gene models are composed of linearly successive exons, all HSPs that break this sequence linearity in either sequence *s_x_* or *s_y_* are discarded.

***Theorem A:*** *the genes G on sequences S must have maintained a certain sequence similarity over their complete length*

At this point, the PAG will contain the vast majority of true gene structure ORFs and similarities, but many biologically irrelevant nodes and edges could be present due to the low threshold used for pairwise similarity. A low threshold is used in order to detect faint and short pairwise similarities too. From here on, edges, nodes and CBGs that belong to the genes of interest will be referred to as true. All others, meaning irrelevant similarities that do represent or link exons of the gene(s), will be referred to as false. We now revert to the coding block concept. A group of *S(S-1)/2* pairwise alignments of exons is identifiable in the PAG as a complete subgraph *K_S_* of *S* nodes and *S(S-1)/2* edges. All *K_S_* subgraphs in PAG are put aside as potential coding block graphs (CBGs). False similarities, so those that do not represent exons of the gene(s),are unlikely to be ubiquitous among all pairwise aligned ORF sets and therefore do not eventuate in *K_S_* subgraphs. This successfully filters out the majority of false pairwise similarities that were detected by using a low score threshold.

At this stage all true CBGs can be correctly retrieved. This is only valid for gene-containing sequences that have a high level of protein similarity over their complete lengths, that lack small exons and lack small CBGs. Regions of little similarity, occurrence of small exons (5~20 nt) and intron-exon gene structures that give rise to small CBGs (<15 nt) will cause some edges or nodes to be absent from PAG.

***Theorem B:*** *small exons are easily overlooked and their detection should be strived for with dedication*

***Theorem C:*** *tiny coding blocks will only occur in cases of small exons because independent events of intron gain are rare*

Three different types of highly connected (but not *K_S_*) subgraphs from PAG can be discerned and each necessitates distinct strategies for attempting completion:

1. graphs with missing edges (*S,P-i* graphs with *i* ∈ {1,2,..*S*-2}) are subjected to more subtle similarity searches (clustalw)
2. complete graphs with missing nodes (*K_S-j_* graphs with *j* ∈ {1,2,..*S*-2}) have their multiple protein alignment transformed into an HMM search profile that is used to scan the ORFs of the *j* sequences that are absent in this subgraph
3. graphs missing edges and nodes (*S-j, P-i* graphs with *i* ∈ {1,2,..*S*-3} and *j* {1,2,..*S*-3}) with high-scoring edges (strong similarity) and ORF node(s) that are not encountered in previous graphs are subjected to both clustalw and HMMER searches

Undetected CBGs can still be hidden inside previously identified complete CBGs as exemplified in Figure 2. In this example, the similarity between the exon on ORF o*_1,2_* and any of the corresponding ORF parts of the other genes is too low, resulting in absence of the complete graph *cb_2_* in the PAG. However, a discrepancy is observed between the size of the extrapolated intersection of cb_1_ and the longer alignment that is identified by a subset of ORFs, as depicted by the dashed parts of *o_3,1_, o_4,1_, o_5_,*_1_. This discrepancy can be described as a complete graph *K_S-1_*, which can subsequently be completed into *cb_2_* (*K_S_*) by recruiting previously identified nodes and edges from the PAG (in this example ORF *o_2,2_*) and additional HMMER searches with an alignment profile to identify a putative existing similar ORF, in this case *o_1,2_*.


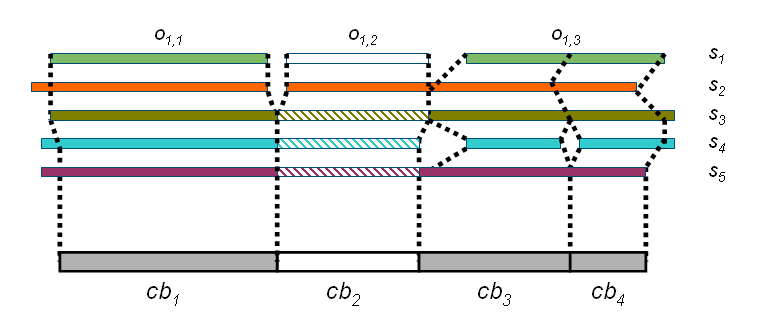


***Figure 2: Schematic representation of a partial gene structure alignment. The existence of the K_S-1_ graph cb_2_ is detected based on length discrepancy of the ORFs in cb_1_.* Cb2 can be completed by recruiting nodes and edges from the PAG (ORF *o_2,2_) and by additional HMMER searches with an alignment profile to detect* ORF *o_1,2_.***

All (nearly) complete subgraphs from PAG are potential coding blocks of the true gene structure. Besides high graph connectivity, a CBG must comply with several other criteria in order to be incorporated in the reconstructed gene structure. The highest scoring CBG is transformed into a GTG that serves as a blueprint of the expected gene tree. All potential CBGs are ordered by their total weight and sequentially added to the reconstructed gene structure when all of the following four criteria are met:

1. CBG corresponds to an extrapolated intersection that is covered by all pairwise alignments (of at least 4 amino acids long). This will filter out CBGs that comprise mostly small, false alignments that by chance coincide into a highly connected subgraph.
2. CBG comprises S nodes. Conservation of neighboring genes in all sequences *S* (complete micro syntheny) is unlikely in distantly related fungal species. This results in true *K_S-x_* graphs that are subjected to a thresholdless HMMER search. A HMMER search that does not yield x nodes is a clear signal for incomplete micro syntheny and these CBGs are not accepted.
3. CBG shows little overlap with previously accepted, higher scoring CBGs (at most 4 amino acids long). Ubiquitous out-of-frame alignments of protein-coding ORFs are likely to coincide into *K_S_* graphs, but these will overlap with and score much lower than the true, in-frame *K_S_*.
4. CBG shows little discrepancy with the absolute and normalized identity scores of the GTG. The GTG_CBG_ representation of the CBG is compared to the GTG itself. The CBG fails addition when its identity score is much lower than that of the GTG or the sum of the differences between all pairwise normalized identity scores is below a certain threshold. Thresholds are defined as a function of the GTG’s identity score (for further documentation, see the main executable abfgp.py). This criterion filters out small, false alignments and spurious alignments introduced by HMMER completed *K_S-x_* graphs.

***Theorem D:*** *topology of all CBGs of genes G should correspond to those of the gene tree graph*

***Theorem E:*** *complete micro syntheny in S fungal sequences does not occur*

A detailed specification of all PAG, CBG and GTG operations and requirements and the exact order in which they are performed to lead to a linearly constrained series of CBGs is provided as documentation in the main executable (abfgp.py).

**From a linearly constrained series of CBGs to a gene structure prediction**

An ordered series of CBGs must be joined by introns to form a valid gene structure. This is performed by assembling four distinct gene structure elements as nodes in graphs: the interface between two adjacent CBGs as potential donor (DSG) and acceptor sites graphs (ASG) and the exterior of the first and last CBG as a graph of potential translational start sites (TSS) and stop codons, respectively. Donor, acceptor and TSS nodes are obtained and scored by a PSSM, stop codon nodes by the end of the ORF. This is explained in more detail by taking introns as an example.

***Theorem F:*** *a general fungal PSSM for gene structure elements is specific enough in individual species*

***Theorem G:*** *splice sites and TSS are canonical*


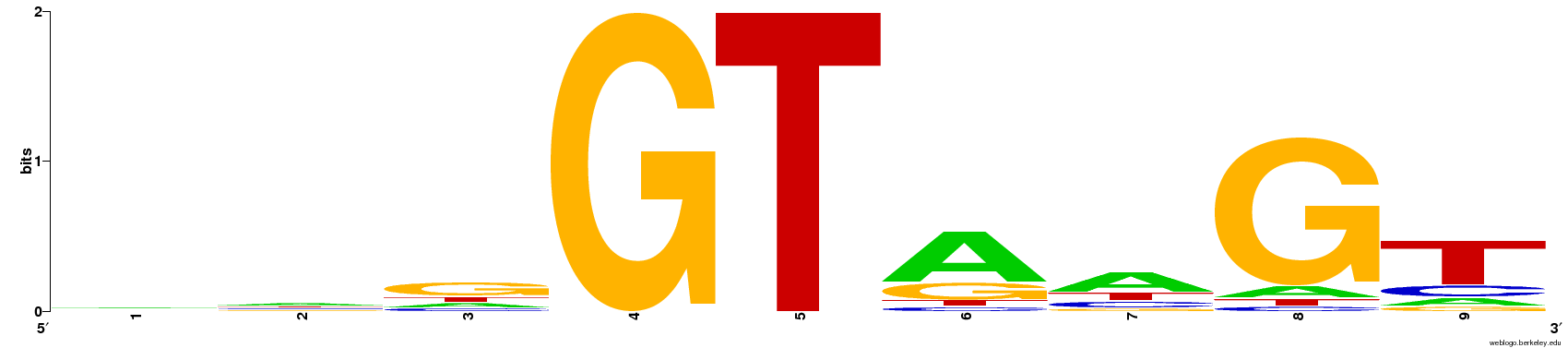


Figure 3a: Position Specific Scoring Matrix representation of the canonical donor site in fungi (WebLogo)


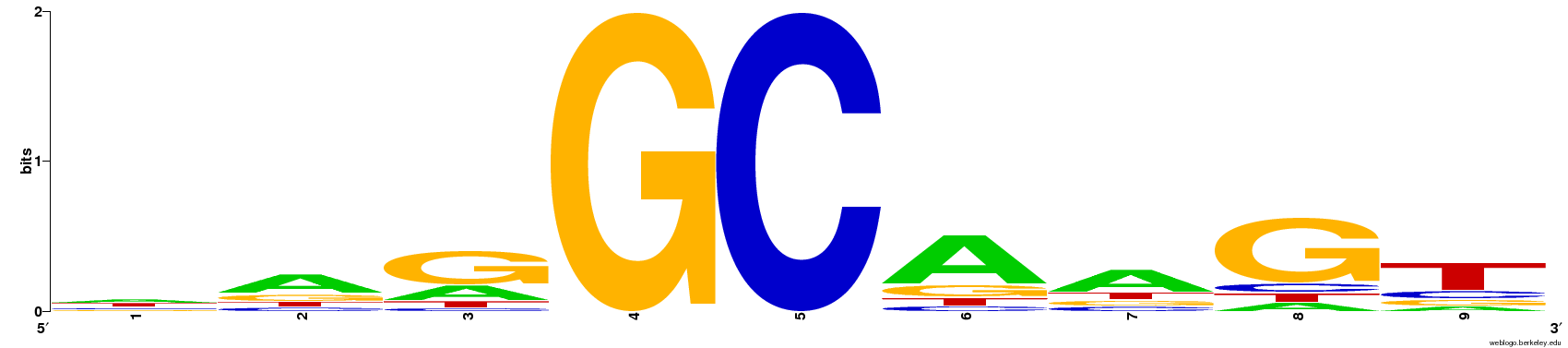


Figure 3b: Position Specific Scoring Matrix representation of the non-canonical GC donor site in fungi (WebLogo)


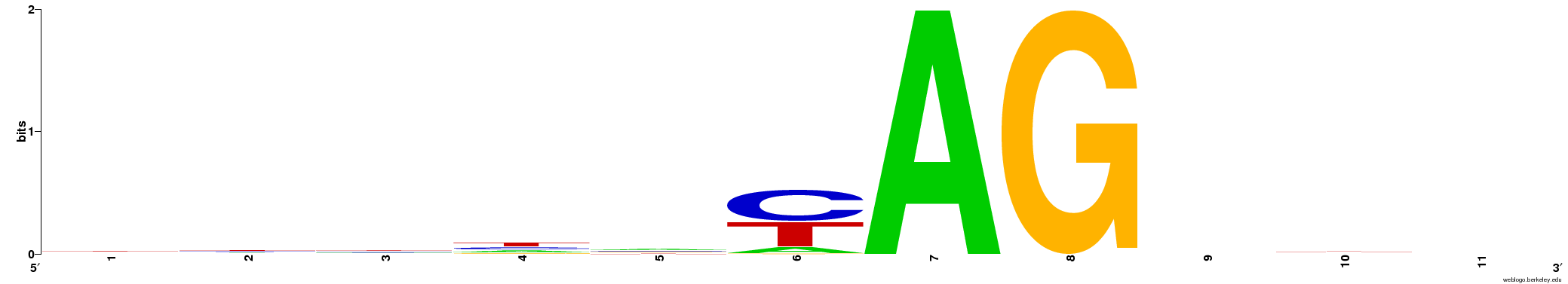


Figure 3c: Position Specific Scoring Matrix representation of the canonical acceptor site in fungi (WebLogo)


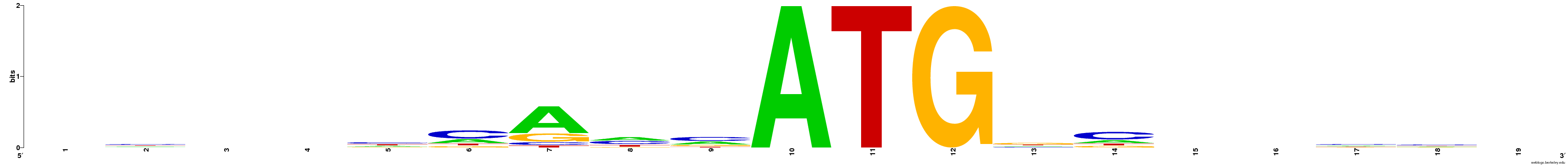


Figure 3d: Position Specific Scoring Matrix representation of the canonical translational start site in fungi (WebLogo)

Intron identification is performed by looking at intron PAPs and at splice sites that are positionally conserved. The nodes in splice site graphs are putative donor- and acceptor sites. These are predicted by a PSSM search of generic fungal splice site (Figure 3a-c; see methods section for further details) with a very low threshold (0.0). Non-canonical donors are allowed (pssm threshold score 3.0); non-canonical acceptor sites and TSS are not taken into account. Edges are created between putative splice sites that are close by in pairwise alignments. This is done by reverse translating the observed amino acid alignment into a DNA alignment. For splice sites, edges are created for sites that are at most *d* =15 nucleotides apart and having identical phases. For TSSs (Figure 3d) and stop codons, the same intrinsic phase identity applies, but no limitation for *d* is set.

***Theorem H:*** *splice site loci are conserved and of identical phase*

Edges are scored according to three different criteria: distance *d* penalizes sites that are nearby but not exactly conserved (d=0), pssm scores of the splice sites and a score that describes the position in the alignments. TSS and acceptor sites are more likely to occur at or around the 5’ boundary of observed protein alignments, whereas stop codons and donors are associated with the 3’ boundary. The alignment, from start to end, is converted into a binary vector in which one is an aligned position and zero is a non aligned position. A position in this vector is scored by applying the Faulhaber’s formula on the ones observed in a window of x aa’s 5’ and 3’ of this position. The positional alignment score is calculated as the difference between the Faulhaber’s 5’ and 3’ sum, divided by the maximum attainable Faulhaber’s sum of x (5’ - 3’ for donors and stop codons, 3’ - 5’ for acceptors and TSSs). *x* is arbitrarily chosen as 10 aa’s. This yields a score ranging from 1.0 to -1.0 for each edge (for more details see the function alignment_entropy() in pacb/pacbporf.py).

Intron PAPs are sought for in case the pairwise alignments in two adjacent CBGs have a shared ORF in one (o_a,x_, o_a,x+1_ ) but not in the other sequence (*o_b,x_, o_c,x+1_*). For absent introns, the nodes are the positions at which the splice sites occur in the sequence that contains an intron. This is referred to as intron projection. PSSM predicted splice sites in the two non-identical ORFs (*o_b,x_, o_c,x+1_*) are combinatorially united in introns. Only introns that result in a continuous pairwise protein alignment on the interface of the CBGs are accepted (at most a gap of 15 nt, so 5 aa). These splice sites are projected onto the identical ORF (o_a_) through the pairwise ORF alignment and subsequently added to their appropriate graphs as projected splice sites, thereby maintaining the properties (pssm score, phase) of their originals. The observed gap is used as the distance *d* variable in edge scoring, thereby rewarding perfect intron PAPs (d=0 nt).

*K_S_* subgraphs in these gene structure element graphs are potential, conserved loci consisting *S* (projected) sites. The total score of these graphs is a weighted mean of the sum of pssm score of the nodes, summed positional score of the edges and the summed weight of the edges (*1/(1-d)*). The highest scoring *K_S_* graphs contain the gene structure elements of the *S* predicted gene structures.

Finally, introns are defined on the interface between two adjacent CBGs by taking the highest scoring pair of *K_S_* subgraphs from DSG and ASG that have matching phases.

In analogy, the TSS of the aligned genes is determined by the highest scoring *K_S_* graph of TSSs of the 5’ exterior of the first CBG. The distance *d_TSS_* is the nucleotide distance between TSS of the same phase in pairwise alignments. A stop codon graph is created for the ORF ends of the posterior CBG. The distance *d_STOP_* is the nucleotide distance between the stop codons of pairwise aligned ORFs.

For each sequence in S, a gene structure is drawn through all the CBGs, using the sites in the highest scoring *K_S_* gene structure element graphs. Inconsistencies in any of these graphs (see below) are tackled when possible, resulting in a single predicted gene structure for each sequence in *S*.

***Theorem I:*** *alternative splicing is of little importance in fungi*

**In search for small exons and CBGs**

The above section describes how graph theory is used to link CBGs into a gene structure. The same graphs can be used as indicators of missing data in their underlying PAG. According to theorem B, small exons are easily overlooked in fungal gene models. Alignment-based evidence can be exploited to search for these small exons in all or a subset of the informants. Graph theory can indicate at this stage missing small exons and thus ORFs in the PAG. This signal can be obtained by close inspection of the interface between two adjacent CBGs and might be:

1. splice site phase discrepancy of the highest scoring K_s_ graph of donor and acceptor sites in the DSG and ASG, respectively
2. identical ORFs for a subset of informants in two adjacent CBGs, combined with K_s-x_ optimal graphs in either DSG or ASG. This indicates that a subset of nodes, and thus splice sites, is not available yet.
3. An optimal K_s-x_ graph in the TSS-graph of the first CBG
4. A poor scoring optimal Ks graph in the TSS-graph. This occurs when a conserved methionine, which is not the TSS, is the optimal Ks graph. Upstream similarity in some but not all informants relative to this site is used as an indicator for not having detected the 5’ delimitation of the gene model yet.
5. Poor distance scores in the frontal TSS or distal stop codon graph. When a subset of sequences *S* has a small first or final exon that is still missing from the PAG, its CBG will be absent too, causing large values for *d_TSS_* or *d*_STOP_ for aligned TSS and stop codons.

Any of these signals triggers an accurate similarity search at the concerning locus in the gene structure graph. First, *ab initio* prediction of several possible tiny exons by combining predicted TSS, donors and acceptors is performed. By evaluating all possible combinations, together with sensitive HMMER searches, various small CBGs are proposed of which the highest scoring option is accepted and incorporated into the gene structure. More detailed information about which steps are exactly undertaken is provided as documentation in the main executable *abfgp.py*.

**Introspection: predicting the correctness of the predicted gene model**

Discrepancies or inconsistencies in any of these gene structure elements *K_S_* graphs is a signal for erroneously predicted gene structure(s). Incorrect gene structures are the result of one or a combination of the following events.

- False negative CBG Theorem A,B,C,D
- Incorrect CBG (having at least 1 incorrect ORF node) Theorem B,C,D
- False positive CBG (having S incorrect ORF nodes) Theorem E
- Splice site discrepancies Theorem C,F,G,H,I
- Erroneous Stop codon (= false or missing final CBG) Theorem A,B,E
- Erroneous TSS (= false or missing first CBG) Theorem A,B,E,F,G
- False positive CBG (having several correct ORF nodes) Theorem I

These events are caused by the invalidity of the postulated theorems that are valid for the majority of gene models. Observed inconsistencies in the concerning graphs are used to label the exons and introns in the predicted gene model as at least doubtful, and possibly incorrect. We applied a simple 2-state predictor:

**ok (True)**  Not a single inconsistency observed; most certainly a correctly predicted gene structure

**doubtful (False)** Inconsistencies or even incorrectness, without any doubt an incorrectly predicted gene structure
