# Supplementary material for: Automated alignment-based curation of gene models in filamentous fungi
Source: BMC Bioinformatics. 2014 Jan 16;15:19. doi: 10.1186/1471-2105-15-19 (PMC3898260; doi:10.1186/1471-2105-15-19)
Supplement: Additional file 3 — Determination of a dataset from ten fungi for benchmarking the ABFGP method. Determination of a dataset of 6,965 experimentally validated genes models from ten fungal genomes for benchmarking the performance of the ABFGP method. [file 1471-2105-15-19-S3.doc]

**Additional File 3: Determination of a dataset from ten fungi for benchmarking the ABFGP method.**

| Fungal species | Source ^1^ |  | Number of unigene sequences | | | | |
| --- | --- | --- | --- | --- | --- | --- | --- |
|  |  | total  sequences | mapped to genome | full-length | identical to annotated gene | ≥4 BDBH informants ^2^ | with alternative splicing ^3^ |
| *Aspergillus flavus* | GI5 | 8,137 | 6,556 | 610 | 211 | 183 | 18 |
| *Cochliobolus heterostrophus* C5 | JGI | 88,751 | 51,553 | 1,162 | 707 | 572 | 12 |
| *Cryphonectria parasitica* | JGI | 19,058 | 15,032 | 1,236 | 708 | 370 | 22 |
| *Dothistroma septosporum* | JGI | 13,184 | 12,956 | 4,556 | 2,902 | 1,829 | 82 |
| *Fusarium verticillioides* | GI8 | 13,350 | 12,104 | 2,070 | 1,498 | 1,154 | 50 |
| *Magnaporthe oryzae* ^4^ | GI6 | 30,708 | 22,741 | 2,833 | 1,622 | 956 | 35 |
| *Neurospora crassa* | GI4 | 14,496 | 13,241 | 1,021 | 792 | 576 | 7 |
| *Nectria haematococca* | JGI | 6,663 | 5,911 | 842 | 522 | 458 | 5 |
| *Trichoderma atroviride* | JGI | 20,489 | 19,147 | 1,314 | 890 | 718 | 10 |
| *Zymoseptoria tritici* ^5^ | JGI | 18,029 | 12,344 | 431 | 243 | 149 | 5 |

^1^ Source of data; Joint Genome Institute (JGI) or The Gene Index Project (GI) with version number

^2^ Informant gene loci recruited as bi-directional best hits (BDBH) to predicted protein sequences

^3^ Gene loci that have full-length unigene evidence for alternative splicing in the part of the messenger RNA that encodes the protein

^4^ Formerly named *Magnaporthe grisea*

^5^ Formerly named *Mycosphaerella graminicola*

The 6,965 gene loci and unigenes that were used in the benchmark analyses are listed in a separate excel file; see ‘access to the method and data’ in the main manuscript.
