# Supplementary material for: Automated alignment-based curation of gene models in filamentous fungi
Source: BMC Bioinformatics. 2014 Jan 16;15:19. doi: 10.1186/1471-2105-15-19 (PMC3898260; doi:10.1186/1471-2105-15-19)
Supplement: Additional file 4 — Benchmarking results of ABFGP performance. Benchmarking results of ABFGP performance on 6,965 experimentally validated gene models from ten fungal species. [file 1471-2105-15-19-S4.doc]

**Additional File 4: Benchmarking results of ABFGP performance on 6,965 experimentally validated genes models from ten fungal species.**

Sensitivity (Sn) and specificity (Sp) of the gene model components (introns, exons, nucleotides) are expressed in percentages. Sn is calculated by true positives / (true positives + false negatives), specificity as true positives / (true positives + false positives) [17]. Gene sensitivity is the percentage of gene models that are predicted without a single error.

| Species |  | *Aspergillus flavus* | *Cochliobolus heterostrophus* | *Cryphonectria parasitica* | *Dothistroma septosporum* | *Fusarium verticillioides* |
| --- | --- | --- | --- | --- | --- | --- |
| # unigenes |  | 183 | 572 | 370 | 1,829 | 1,154 |
| Intron | Sn | 93.73 | 91.63 | 90.50 | 87.93 | 92.19 |
|  | Sp | 96.60 | 97.23 | 97.67 | 95.43 | 98.17 |
| Exon | Sn | 92.39 | 89.59 | 88.36 | 83.88 | 90.38 |
|  | Sp | 98.03 | 98.84 | 98.84 | 98.39 | 98.95 |
| Nucleotide | Sn | 99.25 | 98.86 | 98.65 | 98.66 | 99.26 |
|  | Sp | 98.58 | 99.19 | 97.54 | 99.18 | 98.95 |
| Gene | Sn | 85.2 | 81.3 | 79.2 | 73.6 | 82.1 |

| Species |  | *Magnaporthe oryzae*^1^ | *Neurospora crassa* | *Nectria haematococca* | *Trichoderma atroviride* | *Zymoseptoria tritici^2^* |
| --- | --- | --- | --- | --- | --- | --- |
| # unigenes |  | 956 | 576 | 458 | 718 | 149 |
| Intron | Sn | 91.49 | 90.70 | 93.55 | 94.02 | 85.39 |
|  | Sp | 97.40 | 94.82 | 98.42 | 98.21 | 97.85 |
| Exon | Sn | 89.08 | 87.73 | 92.16 | 91.95 | 84.13 |
|  | Sp | 99.39 | 98.99 | 99.27 | 99.11 | 99.72 |
| Nucleotide | Sn | 98.32 | 98.05 | 99.33 | 99.25 | 98.32 |
|  | Sp | 99.34 | 99.10 | 99.20 | 99.13 | 98.54 |
| Gene | Sn | 81.7 | 76.9 | 85.1 | 83.7 | 74.5 |

^1^ Formerly named *Magnaporthe grisea*

^2^ Formerly named *Mycosphaerella graminicola*
