# Supplementary material for: Automated alignment-based curation of gene models in filamentous fungi
Source: BMC Bioinformatics. 2014 Jan 16;15:19. doi: 10.1186/1471-2105-15-19 (PMC3898260; doi:10.1186/1471-2105-15-19)
Supplement: Additional file 5 — Rank of species providing informant gene loci used for the six re-annotated gene catalogues. Top three and bottom two species that provided the highest number of informants for the re-annotation of the gene catalogues of six fungal species. [file 1471-2105-15-19-S5.doc]

**Additional File 5: Rank of species providing informant gene loci used for the six re-annotated gene catalogues**

## Additional Table – Rank of species providing informant gene loci used for the six re-annotated gene catalogues.

| Informant rank | *Botrytis cinerea* | | *Cladosporium fulvum* | | *Dothistroma septosporum* | | *Mycosphaerella fijiensis* | | *Verticillium dahliae* | | *Zymoseptoria tritici*^1^ | | |
| --- | --- | --- | --- | --- | --- | --- | --- | --- | --- | --- | --- | --- | --- |
| Rank 1 ^2^ | 1,067 Ss | 13% | 498 Ds | 7% | 750 Cf | 9% | 718 Ds | 10% | 1,129 Nh^3^ | 14% | | 1,045 Ds | 13% |
| Rank 2 | 1,357 Fg | 16% | 1,291 Nh^4^ | 17% | 1,199 Zt | 15% | 1,084 An^4^ | 15% | 1,475 Mo^3^ | 18% | | 1,181 Sn^3^ | 15% |
| Rank 3 | 1,396 Fo | 16% | 1,447 Tr^4^ | 19% | 1,341 Mf | 17% | 1,234 Cf | 17% | 1,500 Va | 18% | | 1,247 Pt^3^ | 16% |
| … |  |  |  |  |  |  |  |  |  |  | |  |  |
| Rank 27 | 5,183 Cn | 61% | 5,661 Cn | 75% | 4,987 Ca | 62% | 5,320 Ca | 73% | 5,030 Ca | 60% | | 4,293 Hc | 54% |
| Rank 28 | 5,764 Pg | 68% | 6,137 Pg | 81% | 5,552 Pg | 69% | 5,877 Pg | 81% | 6,993 Pg | 84% | | 4,872 Pg | 62% |

Number and percentage of gene loci eligible for ABFGP for which an informant did not deliver an informant gene locus. Informants are ranked by most to least contributing informants.

^1^ Formerly named *Mycosphaerella graminicola*

^2^ Species with highest (top) and lowest (bottom) rank in providing informant gene loci for ABFGP. Number and percentage of gene models for which this species did not supply an informant gene locus. Species abbreviations used: *Sclerotinia sclerotiorum* (Ss), *Fusarium graminiarum* (Fg), *Fusarium oxysporum* (Fo), *Dothistroma septosporum* (Ds), *Nectria haematococca* (Nh), *Trichoderma atroviride* (Tr), *Cladosporium fulvum* (Cf), *Zymoseptoria tritici* (Zt), *Mycosphaerella fijiensis* (Mf), *Aspergillus nidulans* (An), *Stagonospora nodorum* (Sn), *Pyrenophora tritici-repentis* (Pt), *Magnaporthe oryzae* (Mo), *Verticillium albo-atrum* (Va).

^3^ Based on the fungal phylogeny, the rank of this species was surprising

^4^ Based on the fungal phylogeny, the rank of this species was very surprising
